# Contribution of Gut Bacteria to Liver Pathobiology

**DOI:** 10.1155/2010/453563

**Published:** 2010-07-28

**Authors:** Gakuhei Son, Michael Kremer, Ian N. Hines

**Affiliations:** ^1^Division of Gastroenterology and Hepatology, Department of Medicine, University of North Carolina, Chapel Hill, NC 27599, USA; ^2^Department of Surgery, University of Heidelberg, 69120, Heidelberg, Germany; ^3^Department of Nutrition and Dietetics, College of Human Ecology, East Carolina University, 110 Rivers Building, Greenville, NC 27858-4353, USA

## Abstract

Emerging evidence suggests a strong interaction between the gut microbiota and health and disease. The interactions of the gut microbiota and the liver have only recently been investigated in detail. Receiving approximately 70% of its blood supply from the intestinal venous outflow, the liver represents the first line of defense against gut-derived antigens and is equipped with a broad array of immune cells (i.e., macrophages, lymphocytes, natural killer cells, and dendritic cells) to accomplish this function. In the setting of tissue injury, whereby the liver is otherwise damaged (e.g., viral infection, toxin exposure, ischemic tissue damage, etc.), these same immune cell populations and their interactions with the infiltrating gut bacteria likely contribute to and promote these pathologies. The following paper will highlight recent studies investigating the relationship between the gut microbiota, liver biology, and pathobiology. Defining these connections will likely provide new targets for therapy or prevention of a wide variety of acute and chronic liver pathologies.

## 1. Introduction

Receiving approximately 70% of its blood supply from the portal vein which is the direct venous outflow of the intestine, the liver is continually exposed to gut-derived factors including bacteria and bacterial components. To combat this influx, the liver contains a large number of resident immune cells including macrophages (i.e., the Kupffer cell), lymphocytes, natural killer cells, dendritic cells, and B cells. Together, these immune cell populations in conjunction with other nonparenchymal cells including endothelial cells and stellate cells orchestrate a controlled and organized response to these potentially highly inflammatory factors. However, when normal liver physiology is disrupted and inflammatory cells are activated, gut-derived factors likely augment or exacerbate certain liver diseases leading to enhanced tissue damage and propagation of inflammation. Thus, understanding the mechanisms both of control and of activation by gut-derived factors as well as the functionality of the gut barrier are critical to the development of new therapeutic modalities to treat or prevent acute and chronic liver diseases such as viral hepatitis, alcoholic liver disease, and/or liver cancer. The current paper will provide an overview of gut bacterial populations, gut barrier function, and the potential interactions of gut bacteria with acute and chronic liver disease.

## 2. The Gut Microbiome

The human intestine provides residence to 1 × 10^13^ bacteria, a number which dwarfs the total number of cells in the human body (1 × 10^12^) [1, 2]. Referred to as commensal bacteria, these microorganisms play a crucial role in human physiology and metabolism, providing key metabolic functions during absorption and waste breakdown [3]. Moreover, they also contribute to gut epithelial cell responses including proliferation and differentiation and play a key role in barrier development and function [4]. It is clear, however, that these same luminal contents may also contribute to intestinal pathology in the setting of colitis where immune cell dysfunction, barrier disruption, and/or overgrowth of pathogenic bacterial species have been shown to be involved [5–8]. The following sections will provide a brief overview of the populations present and their proposed functions.

## 3. Populations of Microflora

A number of studies have sought to identify the populations present with estimates of approximately 800 different species [2]. The reasons for these differences in numbers are likely the result of mechanism(s) of detection as early studies used both microscopy and culture where as more recent studies utilize highly sensitive sequencing approaches of 16 S rRNA for identification including 454 pyrosequencing [1]. The predominant bacteria present within the gut are from the genera *bacteroides*, *clostridium*, *bifidobacterium*, *peptostreptococcus*, and *ruminococcus* with *escherichia*, *lactobacillus*, *enterobacter*, and *enterococcus* constituting a minor but significant proportion [1, 9]. Interestingly, the types and proportions of bacteria within the intestine from lower small intestine to distal colon are different, likely regulated both by the microenvironment (i.e., pH and nutrient availability) and the intestinal motility itself [10, 11]. For example, *Escherichia coli*, *enterococci*, and *lactobacilli* account for greater than 50% of cecal bacteria whereas their numbers dwindle to less than 10% in the distal colon. The specific populations present also varies both between individuals and in the same individual during periods of illness or alterations in food intake [9]. Indeed, treatment with antibiotics or acute diarrheal illness have been shown to alter gut microbe densities and populations while alterations in diet (i.e., high fiber) may also cause a minor shift in these populations [12]. Alternatively, specific physiological abnormalities may also contribute to gut bacteria content. For example, pancreatic exocrine insufficiency or vagus nerve defects can reduce antibiotic factors (i.e., pancreatic enzymes) and peristalisis, respectively, leading to increased bacterial growth in the upper gastrointestinal (GI) tract while strictures and adhesions can themselves limit gut content mobility, promoting bacterial overgrowth [13]. Moreover, in animal models, significant differences in gut microbiota have been shown between vendors and as well as in genetically deficient mice. For example, significant differences in both total bacterial load and specific bacterial populations (i.e., segmented filamentous bacteria) were shown between mice purchased from Jackson Laboratories and Taconic Farms [14]. In addition, mice deficient in MyD88, an adaptor molecule associated with numerous innate immune receptors, showed altered gut microbiota in the distal intestine suggesting that immune cell function can interact with and alter luminal bacterial populations [15]. Thus, physiological and pathological changes in intestinal function including alterations in immune cell responses can significantly influence the proportions, quantity, and spatial distribution of bacteria present within the intestine.

The reason for and importance of interindividual differences in gut microbial content in otherwise normal individuals is less clear [16]. The human microbiome project has been initiated by the National Institutes of Health in the United States to characterize the bacterial populations present both within healthy and diseased individuals from all over the world. Early results demonstrate what appear to be a core group of bacteria which is shared by most individuals though the vast majority of bacteria are still heterogeneous among different individuals [16]. Together, it is clear that significant diversity exists among intestinal bacterial species between individuals and that certain environmental, physiological, pathological, or therapeutic interventions can modulate these populations.

## 4. Function of the Gut Microflora

As noted above, intestinal microbiota are referred to as commensal as they coexist without initiating inflammatory or infectious responses. It is becoming clear that these same bacteria provide at least three key functions to the mammalian intestine including epithelial cell health, nutrient metabolism and breakdown, and indirect mucosal defense against pathogenic bacterial strains. Perhaps the most easily understood function of these bacteria is their contribution to metabolism and nutrient breakdown. Commonly represented genera of bacteria within the human intestine are known to express key polysaccharide metabolizing enzymes capable of breakdown of routinely consumed sugars including cellulose, pectins, and gums [17]. These same bacteria also affect gut barrier function. Indeed, these nonpathogenic bacteria compete for nutrients and adherence with other pathogenic bacteria. A good example of this effect is seen in experimental animals as well as patients receiving high-dose antibiotic treatment where reductions in normal gut flora allow for overgrowth of pathogenic bacteria including *Clostridium difficile* [18, 19]. The generation of germ-free mice has further confirmed the importance of commensal bacteria in health and barrier function. Intragastric infection of germ-free mice with *E. coli* O157:H7 resulted in rapid intestinal colonization and morbidity associated with glomerular toxicity, a response not observed in conventionalized mice [20]. Again, overgrowth of pathogenic bacteria in a germ-free host is the likely cause for these findings though alterations in barrier integrity or immune cell development, localization, or responsiveness may also contribute. 

Finally, intestinal microbiota also influence epithelial cell health and function. When biota are absence as in the germ-free mice, intestinal epithelial cells are underdeveloped [21, 22]. As discussed above, gut flora play a key role in metabolism of complex sugars. During this process, fermentation of sugars forms a number of short chain fatty acids including proprionate, acetate, and butyrate among others [1]. Interestingly, intestinal epithelial cells derive a large percentage of their metabolic fuel from these products, specifically butyrate. *In vitro* butyrate administration to cultured intestinal epithelial cells promotes their survival, differentiation, and proliferation thereby supporting barrier integrity [23]. Together, these studies and numerous others support a specific and tightly regulated role for enteric bacteria in metabolism, defense, and barrier integrity within the intestine.

## 5. Gut Barrier Function and Dysfunction

In as much as gut bacteria contribute to normal gut physiology, their presence poses a continuous risk for systemic infection [6]. The direct physical barrier against bacterial translocation is complex. Mucous producing goblet cells secrete a thick layer of polysaccharide called mucin which coats the intestinal epithelial surface and provides a physical barrier suppressing epithelial-bacteria contact. This layer constitutes in part the unstirred layer covering the intestinal epithelium, slowing the movement of solutes and bulk fluid through the barrier [24]. The specific importance of mucins in protection against intestinal inflammation can be seen Muc2-deficient mice [25]. Mice lacking Muc2 show increased susceptibility to dextran sodium sulfate-mediated colitis. Moreover, in humans, polymorphisms in Muc3A correlate with increased frequency of ulcerative colitis [26]. Thus, the mucous layer is a critical component of the intestinal barrier limiting direct access to the intestinal epithelium. 

Below the mucous layer resides the intestinal epithelial layer. Organized in a crypt and villus arrangement to increase surface area, intestinal epithelial cells (IECs) are held together by a series of cell-cell protein interactions which tightly regulate paracellular solute movement [27]. Nearest the surface of the epithelial cell, claudins interact with intracellular support provided by zonula occluding (ZO-1) and F-actin forming the tight junction. Secondary to the tight junction exists the adherens junction. E-cadherins attach cell membranes, supported by intracellular catenins *α* and *β*. Near the basolateral surface, the desmosome exists consisting of desmogleins and desmocollins anchored to intracellular keratin by desmoplakin [6]. Together, these three structures support strong epithelial cell contacts and prevent paracellular movement of large molecules and bacteria to the underlying tissue. 

Despite this tight and redundant barrier, gut bacteria are continuously sampled by the underlying lamina propria immune cells. Indeed, this underlying layer contains a large population of lymphocytes, dendritic (DC) cells, and neutrophils which serve to intercept invading pathogens and modify the underlying immune response to commensal bacteria populations. DCs extend projections through the epithelial layer, sample enteric bacterial antigens, and present them to underlying lymphocytes, thereby priming the immune system in case of barrier dysfunction [28, 29]. T cell development is then regulated by the production of key cytokines produced by myeloid cells where IL10 principally contributes to T regulatory cell development and maintenance and suppression of inflammation [7]. However, other very recent studies indicate that direct interactions of gut-derived antigens signaling through Toll-like receptor (TLR) 4 on CD4^+^ T cells contributes to their regulatory development and function [30]. In summary, the epithelial barrier and underlying immune cells work together to protect against translocation, inflammation, and systemic infection. 

When this complex barrier and/or underlying immune cell network is damaged or disrupted, intestinal inflammation, tissue damage, and absorptive dysfunction result. Key mechanisms in this disruption have been elucidated and involve immune cell dysregulation, pathogenic bacterial overgrowth, and/or primary barrier dysfunction. Absence of interleukin (IL) 10, a key regulatory cytokine, is known to lead to spontaneous intestinal inflammation in a gut bacteria-dependent manner [31]. Likewise, reconstitution of severe combined immunodeficient mice or recombinase activating gene 1 deficient mice with naïve CD4^+^CD45RB^Hi^ positive T cells results in significant intestinal inflammation and barrier disruption again due to dysregulation of lymphocyte responses [32]. Infection of germ-free mice with certain pathogenic strains of *Campylobacter* can also lead to mild to moderate intestinal inflammation [33]. Similar correlations have been established in human inflammatory bowel disease (IBD) where increased proportions of *E. coli* are noted and can be correlated with the severity of disease [34]. Finally, and as discussed above, alterations in mucin production predispose the intestine to inflammation and bacterial translocation. It is likely, however, that in mice and humans, a combination of factors exist which alter the intestinal barrier, enhance bacterial translocation, and promote intestinal inflammation. Nevertheless, key participants in the regulation of gut barrier function have been established and serve as targets for therapeutic intervention.

## 6. Gut-Liver Interactions in Liver Disease

As the preceding discussion has indicated, gut barrier function is critical to prevent inflammation of the underlying mucosa and submucosa. Receiving ~70% of its blood supply from the intestine through the portal circulation, the liver, much like the intestine, is also exposed to gut-derived factors including bacteria and bacterial products and thus must be prepared to handle these potential systemic pathogens. To accomplish this task, the liver contains a large number of immune cells, of both the innate and adaptive immune systems which participate both in tolerance and inflammation within the liver. The following section will provide a brief overview of these immune cell populations, including their locations, proportions, and general functions.

## 7. Hepatic Immunology: An Overview

Perhaps the most characterized of these immune cell populations is the Kupffer cell (KC), the resident hepatic macrophage. Making up approximately 4% of the total hepatic cell population and 80–90% of all tissue macrophages, KCs are well known for the ability to engulf bacteria and respond to bacterial antigens including lipopolysaccharide (LPS) derived from gram negative bacteria such as E. coli [35]. Through the expression of TLR4 and CD14, KCs are able to efficiently take up endotoxin and phagocytose portally delivered bacteria while also contributing significantly to inflammation and tissue damage through the production of tumor necrosis factor alpha (TNF*α*) and reactive oxygen intermediates in a wide variety of acute and chronic liver disease [36, 37]. Alternatively, KCs may serve to tolerize the immune response through antigen presentation and concomitant nitric oxide and prostaglandin production [38]. It is clear given their sheer numbers that KCs are an important component of the innate immune response of the liver. 

Working in concert with these resident macrophages are DCs. DCs are also capable of engulfing particles including bacteria but play a key role in antigen presentation, cytokine production (i.e., IL4 and 12 production), and T and B cell development and reactivity [39]. DCs may also promote natural killer (NK) and natural killer T (NKT) cell activation via IL12 production and accelerate tumor cell clearance and their reduced numbers in the hepatitis C virus (HCV) infected liver may enhance HCV infectivity and carcinogenicity [40, 41]. 

Complimenting the functions of KCs and DCs are those of natural killer (NK) cells. NK cells express specific receptors (NK1.1, NKG2D in mice; NKp46, CD56, and CD57 in humans) and produce large amounts of perforin and granzyme B in addition to immunomodulatory factors such as interferon gamma (IFN*γ*) and TNF*α* upon activation [42, 43]. NK cells are particularly responsive to malignant or infected cells while also potentially contributing to transplant rejection and autoimmunity [44, 45]. Indeed, depletion of NK cells promotes graft survival while their activation suppresses cancer cell survival and proliferation. Moreover, NK cells suppress fibrogenesis through direct killing of hepatic stellate cells (HSCs) in an NKG2D and IFN*γ* dependent manner [46]. 

Bridging the gap between innate and adaptive immunity is the natural killer T (NKT) cell. Expressing receptors for both innate (NK1.1, CD49b, CD56, and CD57) and adaptive (T cell receptor) immune cells, NKT cells represent an important source of IFN*γ* and IL4 within the liver [47]. A large proportion of hepatic NKT cells recognize antigens presented through the MHC Class I-like receptor CD1d, rely heavily on IL12 and IL15 for survival and activation, and contribute both to the regulation of T helper (T_h_) cytokine production and to acute and chronic liver injury through cytokine production and Fas expression [48–50]. Much like NK cells, activation of NKT cells results in tumor cell clearance while also contributing to early alcohol-induced liver injury [50, 51]. 

Fulfilling the adaptive immune functions within the liver are a large population of traditional CD4^+^ and CD8^+^ lymphocytes. Constituting approximately 35% of the hepatic lymphocyte population, these cells play a key role both in antigen recognition and in tolerance [52]. Accumulation and/or survival of hepatic T cells is associated with worse fibrogenesis while their early accumulation in the ischemic liver is a known trigger for neutrophil infiltration and tissue damage [53, 54]. CD8^+^ T cells contribute to stellate cell activation during carbon tetrachloride induced fibrosis and directly damage hepatocytes in the HCV-infected liver in an antigen specific manner [55, 56].

**Figure 1:**
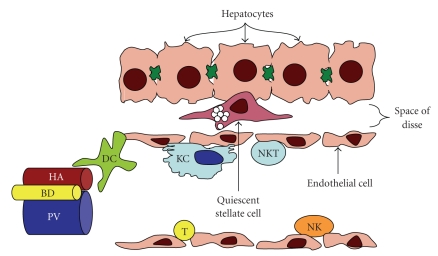
Diagramatic representation of the liver sinusoid. HA; hepatic artery. BD; bile duct. PV; portal vein. DC; dendritic cell. KC; Kupffer cell. NKT; natural killer T cell. T; T cell. NK; natural killer cell.

In summary, and as is shown in Figure 1, the liver provides residence to a large and heterogeneous population of immune cells, each with specific functions of protection, tolerance, and/or inflammation. It is this third aspect, during inflammatory responses or chronic injury, where the function of hepatic immune cells is perhaps most interesting and extensively studied. And of even greater interest is the potential impact which gut-derived factors may have on this process. As noted earlier, the liver is a unique position where its normal function to sample, metabolize, synthesize, and/or degrade both absorbed and circulating products also places it in potential direct contact gut-derived bacteria and bacterial antigens. And previous studies would suggest that a connection exists as either small intestinal bacterial overgrowth or infection with helicobacter alone contributed to hepatic pathology including increased serum alanine aminotransferase release and inflammatory cell recruitment [57–59]. Likewise, experimental colitis models in rodents and inflammatory bowel disease in patients were associated with periportal inflammation similar to that seen in primary biliary cirrhosis and primary sclerosing cholangitis, respectively, suggesting that gut-derived factors likely activate inflammatory processes within the liver [60, 61]. The following sections will highlight the current knowledge regarding the influence of gut-derived factors on hepatic biology and pathobiology, focusing on several important mechanisms of liver injury.

## 8. Gut Bacteria and the Undamaged Liver

The contribution of gut bacteria to the formation of the hepatic immune system has not been intensively investigated. It is clear from the previous discussion that the liver contains a large number of immune cells though the specific mechanisms governing their localization is not well understood. Crispe and colleagues identified TLR4 as a potential indirect regulator of activated CD8^+^ T cell trapping within the murine liver suggesting a potential interrelation between the gut antigens, specifically endotoxin, and liver lymphocyte populations [62]. Recent studies from our laboratory were directed at better understanding the connection between gut bacteria and resident hepatic immune cells. Using germ-free C57Bl/6 wild type mice or specific pathogen free (SPF) mice, we demonstrated that gut bacteria have little effect on the proportions of or total numbers of lymphocytes (CD4, CD8, NK, or NKT cells) or macrophages present within the murine liver. Moreover, examination of serum alanine aminotransferase levels and basal expression of key inflammatory cytokines including TNF*α* and antiinflammatory cytokines (i.e., IL10) were not different between germ-free and SPF mice (Son and Hines, unpublished observation). Finally, analysis of basal hepatocyte proliferation revealed no substantial differences between these groups. Together, these data demonstrate that resident hepatic immune cells and hepatocytes themselves are not overtly affected by normal gut-derived antigen exposure.

While gut bacteria do not significantly affect liver physiology or immune cell populations, its potential to initiate and/or propagate liver injury has been investigated. For example, experimental damage of the intestine with dextran sodium sulfate (DSS) leads to periportal liver inflammation likely the result of increased gut bacterial delivery to the liver [63]. Similarly in patients with ulcerative colitis there is often evidence of primary sclerosing cholangitis including significant periportal inflammation [64]. Thus, it is clear that a relationship exists between gut barrier function and secondary liver inflammation. The following sections will provide a review of the current understanding of gut-derived factors in a number of primary liver pathologies.

## 9. Alcoholic Liver Disease (ALD)

Perhaps the best characterized model of liver disease which is nearly completely dependent on gut-derived factors for its pathogenesis, chronic alcohol consumption remains an important clinical problem alone and in combination with other liver diseases [65]. Early clinical studies revealed increased plasma endotoxin levels following acute ethanol exposure in patients with and without chronic liver disease suggesting that ethanol could potentially alter gut barrier function [66]. Experimental studies confirmed these findings identifying the ability of alcohol to injure the rodent liver through augmentation of gut-derived bacterial translocation (specifically increased periportal levels of LPS) and specific activation of Toll-like receptor 4 on KCs [67–70]. Indeed, sterilization of the gut, depletion of KCs, or mutation in TLR4 caused a near complete inhibition of ethanol-induced liver injury as characterized by serum alanine aminotransferase release, inflammatory cell infiltration, and hepatocellular lipid accumulation. This central role for endotoxin in the pathogenesis of early ALD could not be argued though the mechanism by which ethanol altered gut permeability was less clear. Consumed ethanol is rapidly absorbed by the upper GI tract with near complete absorption occurring by the mid-jejunum. However, the majority of bacteria are held, as described earlier, within the cecum and upper large intestine. Careful studies have demonstrated the ability of ethanol to suppress endotoxin uptake by KCs and the function of acetaldehyde, the principle by-product of ethanol metabolism, to directly interfere with tight junction and adherens junction support [71, 72]. Indeed, absorbed circulating ethanol directly inhibits phagocytosis of macrophages including KCs thereby limiting LPS clearance while acetaldehyde promotes ZO-1 dissociation from occludin and E-cadherin adherence to *β*-catenin [73, 74]. Further compounding these effects is the ability of gut bacteria to metabolize ethanol and thus increase the luminal concentration of acetaldehyde and the potential for bacterial overgrowth to occur in ethanol consuming individuals [71, 75]. Thus, gut bacteria play a key role in early alcohol-induced liver injury both through the metabolism of ethanol and through the activation of key hepatic innate immune cell populations. Key questions remain, however, including the net effect of ethanol on the gut microbiota and the influence of gut-derived antigens on the progression of ALD, specifically fibrogenesis. 

## 10. Nonalcoholic Fatty Liver Disease (NAFLD)

NAFLD continues to increase in westernized countries. Once considered a benign event, accumulation of lipid within the liver is a known risk factor for the development of inflammation and fibrosis and a component of metabolic syndrome, insulin resistance, and obesity [35]. The mechanism(s) underlying the development of NAFLD are not well understood, though obesity is a key risk factor. Numerous studies have implicated liver TNF*α* as a potential regulator of its development and TNF*α* levels are elevated in several models of NAFLD in rodents and in patients with NAFLD [76, 77]. Likewise, deficiency in leptin is known to result in hepatic lipid accumulation in conjunction with peripheral obesity [48, 78]. The potential influence of gut bacteria on the development of hepatocellular steatosis has been postulated. Models of bacterial overgrowth have shown promise as an initiator of fatty liver and patients with NAFLD present with upper intestinal bacterial overgrowth and enhanced intestinal permeability [79, 80].Consistent with gut-derived endotoxin mediating these effects, TLR4-mutant mice showed reduced lipid accumulation following feeding a high-fructose diet or methionine-choline deficient diet when compared to their TLR4-wild type controls suggesting that LPS may contribute to disease progression [81, 82]. Studies from our laboratory and others have confirmed enhanced sensitivity of the liver to endotoxin treatment suggesting that hepatic immune cells, likely KCs, are primed to respond with increased production of TNF*α* and IL12 [49]. Similarly, TLR9 signaling may also promote NAFLD. Miura and colleagues demonstrate a significant reduction in hepatic lipid accumulation in choline deficient, amino acid defined diet in TLR9-deficient mice when compared to their wild type controls, a process which appears to involve TLR9-dependent Kupffer cell activation and subsequent production of IL1*β* [83]. Thus, both cell wall components and DNA derived from bacteria could be involved in the disruption of normal hepatocyte function in the setting of NAFLD.

While the above mentioned studies have focused on the direct interactions of gut-derived bacteria and bacterial products, the potential influence of certain metabolites of gut bacteria including short-chain fatty acids on hepatic energy homeostasis has not been thoroughly explored. It is clear that gut bacteria provide important metabolic functions, metabolizing complex sugars into short-chain fatty acids including proprionate and acetate, two molecules which are key sources of energy for the liver and muscle, respectively [1]. This important function is reinforced by findings in germ-free mice which are substantially leaner than their colonized counterparts. The specific mechanism by which SCFAs affect energy balance is not entirely clear though increased delivery of short chain fatty acids to the liver and the periphery could disrupt normal metabolic processes, specifically reducing glucose utilization and promotion lipid storage. However, high levels of propionate derived from fiber metabolism inhibit cholesterol and fatty acid biosynthesis in rodent livers [84]. Moreover, high-fat diet feeding supplemented with fermentable dietary fiber reduces plasma endotoxin levels when compared to high-fat diet feeding alone suggesting that fiber may affect the gut microbiota and potential influence intestinal barrier function [85–87]. Thus, the net effect of the gut microbiota on nutrient and energy balance is complex and warrants further investigation as it is likely to contribute, in addition to the direct effects on hepatic cells, to hepatic lipogenesis, endotoxemia, and NAFLD.

## 11. Ischemic Liver Injury

Liver transplantation remains the primary treatment for a number of chronic liver diseases including cirrhosis. Organ preservation and associated ischemic tissue damage represents an important determinant of graft survival. Critical mechanism(s) in reperfusion injury have been delineated and include early CD4^+^ lymphocyte recruitment, KC activation and subsequent TNF*α* production, and later neutrophil recruitment and hepatocellular damage [54, 88, 89]. The contribution of gut bacteria to these processes has not been thoroughly investigated though it is clear that bone marrow derived cells and Kupffer cells and TLR4 are critical for early tissue damage following warm ischemia and reperfusion injury [88, 90–92]. It is also well established both experimentally and perhaps more so clinically that intestinal injury occurs during liver surgery and transplantation. Congestion of the portal vein due to clamping, even intermittently during transplantation or resectional surgery, reduces barrier integrity and promotes bacterial translocation [93]. Supporting the impact of intestinal microbiota directly, gut sterilization suppresses transplant-induced liver injury in rodents and reduces the incidence of sepsis early following transplantation in patients suggesting that intestinal microbiota are involved in these processes [94, 95].

## 12. Hepatocellular Carcinoma

The occurrence of hepatocellular carcinoma (HCC) continues to rise clinically. Both environmental and genetic factors have been implicated in its initiation. While susceptibility genes have been identified and range from immune cell markers to traditional tumor suppressor genes, the potential environmental cues are less well established [96]. It is clear that certain hepatotoxins including nitrites, organochlorine compounds, and aflatoxin contribute significantly to its development as do preneoplastic injuries associated with HCV infection [97–99]. The particular contribution of gut bacteria to the development and progression of HCC remains somewhat unclear. Gut bacteria convert intestinal nitrates to nitrites and nitrosoamines which are linked to colorectal cancer [1]. Moreover, recent studies have highlighted the ability of helicobacter hepaticus to promote aflatoxin-induced HCC in mice, a process involving inflammatory and proproliferative cytokine production [100]. These studies correlate well with findings in humans where *helicobacter sp.* can be both cultured from the liver and shown to be in greater quantities in the intestine [58, 101, 102]. Thus, bacterial metabolism of ingested materials and byproducts as well as inflammatory responses to the bacteria themselves likely play critical roles in liver cancer development and/or progression.

## 13. Liver Fibrosis

Chronic liver injury arising from a number of etiologies ranging from chronic ethanol consumption to viral infection is associated with increased risk for the development of fibrosis, cirrhosis, and liver failure [103]. It is clear from a large body of experimental work that repeated and continuous hepatocellular damage leads to the activation of HSCs and their production of key extracellular matrix proteins, specifically Type I fibrillar collagens. A number of studies have implicated immune cell activation, specifically macrophages and lymphocytes, in the initiation and propagation of disease [55, 104, 105]. Indeed, depletion of T and B lymphocytes or macrophages significantly reduced carbon tetrachloride-induced liver injury [104, 105]. The specific role which gut-derived antigens play in the setting of fibrotic liver disease has recently been examined [104]. Sterilization of the gut prevented both toxin-induced and cholestasis-induced hepatic fibrosis [104]. Further characterization revealed a critical role for gut-derived endotoxin as mice deficient in either CD14 or Toll-like receptor 4 were protected from cholestasis-induced fibrogenesis [104, 106]. Indeed, it appears that toxin-induced tissue injury, either by carbon tetrachloride or cholestasis, leads to increased portal delivery of endotoxin, activation of hepatic macrophages, induction of growth factor production, specifically transforming growth factor beta, and subsequent HSC activation [104, 106]. Moreover, recent studies also highlight the ability of endotoxin to directly activate HSCs further amplifying the fibrogenic response in these models [107, 108]. Together, it is clear that gut microflora, and specifically gram negative bacteria, contribute to fibrosis induction and progression experimentally. 

The process of fibrogenesis may itself promote bacterial overgrowth and barrier dysfunction. Cirrhotic patients are at increased risk for spontaneous bacterial peritonitis in conjunction with reduced blood flow through the portal vein, intestinal vascular congestion, and barrier leakiness [109, 110]. Additionally, fibrosis and associated defective liver function itself may promote changes in bacterial populations, intestinal motility, and nutrient absorption and availability. For example, decreased bile acid production by the cirrhotic liver is associated with bacterial overgrowth [111]. Indeed, bile acids play a critical role in regulation of bacterial survival within the intestine [112]. Bile acids are directly, though weakly, bacteriocidal but are capable of activating specific bile acid receptors including farnesoid X receptor which regulates the expression of key bacteriocidal genes including inducible nitric oxide synthase and IL18 within the intestine [113]. Cirrhosis also limits small intestine motility which has been associated with bacterial overgrowth [110]. Finally, decreased absorption increases nutrient availability throughout the small and large intestine further enhancing bacterial growth [114]. Together, it is clear that hepatic fibrosis is critically regulated by gut-derived antigens and that cirrhosis itself may influence the populations of bacteria present within the intestine promoting a positive feedback loop perpetuating tissue injury and fibrogenesis.

## 14. Autoimmune Liver Diseases

Autoimmunity is associated with several forms of chronic liver damage including autoimmune hepatitis, primary biliary cirrhosis, and primary sclerosing cholangitis. The initiating events in these diseases are not well understood though it is clear that antibody formation to self antigens is key to the development. The influence of the gut microbiota on these disease processes again has not been thoroughly investigated though some connections have been suggested. 

Autoimmune hepatitis (AIH) accounts for approximately 20% of chronic hepatitis in Caucasians and is characterized by hypergammaglobulinemia and liver-directed autoantibodies resulting in large hepatic lymphocytic infiltrates [115]. Activation of hepatic T lymphocytes with the plant lectin Concanavalin A leads to the expression of key T helper cytokines including IL4 and IFN*γ*, macrophage activation, neutrophil recruitment, and hepatocellular injury similar to that observed in autoimmune hepatitis [116]. It is clear from this model system that interruption in the early expression of either IL4 or IFN*γ* or disruption in Fas-FasL signaling protect the liver from this T cell-mediated tissue injury [117–121]. The contribution of gut bacteria to this response has not been thoroughly investigated. Previous studies have demonstrated the contribution of TLR4 signaling to the trapping of CD8^+^ T cells within the murine liver [62]. More recently, TLR9 was shown to contribute to the homing and activation of hepatic NKT cells, a process dependent on KCs and IL12 [51]. Similarly, TLR4 expression on intestinal CD4^+^ T cells contributed to the induction of T regulatory cells and suppression of colitis resulting from absence of IL10 [30]. Thus, T cells appear to be capable of responding to conserved antigens such as endotoxin directly and this cascade likely contributes to their responsiveness within the liver. Consistent with this notion, recent studies in our laboratory demonstrate the necessity of gut-derived bacteria during ConA-induced T cell-mediated hepatitis (Son and Hines, unpublished observation). Absence of gut bacteria significantly reduced ConA-induced liver injury in the absence of major alterations in resident T cell number or activation. Indeed, absence of gut bacteria-reduced early IFN*γ* and IL4 production, and later eosinophil recruitment and hepatocellular apoptosis. Together, these studies suggest that gut-derived products regulate, either directly or indirectly, T cell function within the liver. Further study will be required both in animal models and in patients with AIH to more specifically delineate the mechanism governing these responses. 

Similar to AIH, primary biliary cirrhosis affects approximately 40 per 100,000 people in the United States and is a consequence of immune cell activation and directed damage to cholangiocytes, specifically intrahepatic bile ducts with nearly 95% of patients presenting positive for antimitochondrial antibodies [115]. Progressive intrahepatic biliary tract damage promotes bile acid buildup, stellate cell activation, and hepatic fibrogenesis with failure occurring in 26% of patients within 10 years of diagnosis with liver transplantation constituting the primary treatment. Thus, understanding the factors which may promote or exaggerate this process are needed. Studies by Hopf and others detailed an association of *E. coli* rough form and the presence of PBC in patients as healthy individuals rarely show measurable levels of this bacterial subspecies. Moreover, they demonstrated the presence of lipid A within the liver of PBC but not healthy control patients further demonstrating the presence of bacteria within the liver [122]. Given this association, further study is warranted to determine if modulation of gut microbiota, particularly E. coli subpopulations might aid in the treatment of this complex disease. 

Very similar to PBC, primary sclerosing cholangitis can be described as a progressive autoimmune disease process leading to destruction of intrahepatic and extrahepatic bile ducts, inhibition of bile acid secretion, toxin buildup, and chronic hepatocellular injury [123]. Interestingly, as mentioned previously [64, 115], a large number of patients (~75%) show signs of inflammatory bowel disease suggesting potential interactions of the gut and liver and/or common pathological causes (i.e., autoimmune disorders, defective immune cell regulation). Experimental models of inflammatory bowel disease have been associated with periportal inflammation suggesting potentially that gut factors may initiate the response in the absence of underlying immune cell dysfunction [124]. Further examination of the gut microbiota in conjunction with PSC may unlock new information into the mechanisms of PSC and aid in therapeutics development.

## 15. Viral Hepatitis

Hepatitis arising primarily from HCV infection represents the leading cause of liver disease in the world [125]. Indeed, hepatitis B and C viral infections account for 75% of the cases of liver disease worldwide [126]. The pathogenesis, particularly of HCV infection, is complicated and involves primary hepatocyte infection, disruption of immune cell responses including inhibition of endogenous antiviral responses and activation of adaptive immunity including antigen specific CD8^+^ T cell recruitment [126]. The contribution of gut-derived antigens to the pathogenesis of viral hepatitis has not been explored. HCV infection is associated with a number of hepatic diseases from hepatocellular lipid accumulation to stellate cell activation, immune cell recruitment, and cancer development. To this third end, very recent studies by Machida and colleagues identify an important connection between ethanol consumption, viral infection, TLR4 signaling, and carcinogenesis within the murine liver. Indeed, TLR4 signaling promotes Nanog/CD133 production and promotes ethanol/HCV-induced hepatic tumor formation [127]. Future study in this complex system using rodent models is warranted to better understand the overall impact of gut bacteria to the multiple pathologies present. Indeed, it could be that gut-derived antigens serve to prime hepatic innate immune cells to produce important antiviral cytokines including IFN*γ* while also promoting hepatic T cell function and responsiveness. Further study is warranted to dissect out the potential multiple pathways of involvement of gut-derived antigens in this complex injury scenario.

## 16. Concluding Remarks

**Figure 2:**
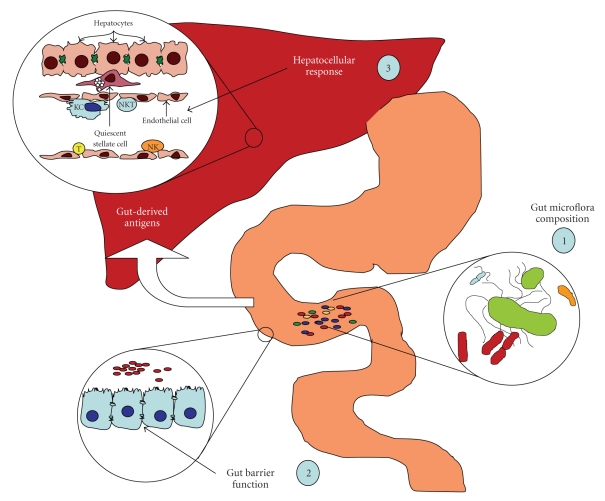
Diagram of the points of future study/intervention to treat or prevent various liver diseases. Discussed in summary section of the text.

From the above discussion, it is clear that gut bacteria contribute to normal intestinal epithelial cell biology and function while also contributing substantially to the breakdown of complex sugars in the diet. It is also evident that these same bacteria, in the absence of appropriate immune cell regulation or when gut barrier function is impaired, contribute significantly to intestinal inflammation and damage. Likewise, these same antigens, when delivered to the liver, contribute significantly to various acute and chronic liver diseases through activation of both innate and adaptive immune responses and wound healing processes. Thus, modulation of the gut microbiota may represent a new avenue for therapeutic intervention to treat or prevent a variety of liver diseases. As detailed in Figure 2, key questions remain, however, including (1) what are the specific populations of bacteria present within the intestine and can these be correlated with or used as a screening tool for the progression of liver disease, (2) how do different microbiota populations influence gut barrier integrity, and (3) what are the cell-specific effects of gut-derived antigens within the injured liver (i.e., KC, stellate cell, T cell, endothelial cell, etc.) and does the type of injury influence their effects (i.e., ischemic damage versus viral infection). Future studies directed at these questions will provide important new information into the connection between the gut microbiota and liver disease and likely contribute to new therapies for or predictors of liver pathobiology.

## References

[1] Guarner F, Malagelada J-R (2003). Gut flora in health and disease. *Lancet*.

[2] O’Keefe SJD (2008). Nutrition and colonic health: the critical role of the microbiota. *Current Opinion in Gastroenterology*.

[3] Musso G, Gambino R, Cassader M (2010). Gut microbiota as a regulator of energy homeostasis and ectopic fat deposition: mechanisms and implications for metabolic disorders. *Current Opinion in Lipidology*.

[4] Rawls JF, Samuel BS, Gordon JI (2004). Gnotobiotic zebrafish reveal evolutionarily conserved responses to the gut microbiota. *Proceedings of the National Academy of Sciences of the United States of America*.

[5] Hering NA, Schulzke J-D (2009). Therapeutic options to modulate barrier defects in inflammatory bowel disease. *Digestive Diseases*.

[6] Turner JR (2009). Intestinal mucosal barrier function in health and disease. *Nature Reviews Immunology*.

[7] Murai M, Turovskaya O, Kim G (2009). Interleukin 10 acts on regulatory T cells to maintain expression of the transcription factor Foxp3 and suppressive function in mice with colitis. *Nature Immunology*.

[8] Garrett WS, Gordon JI, Glimcher LH (2010). Homeostasis and inflammation in the intestine. *Cell*.

[9] Moore WEC, Moore LH (1995). Intestinal floras of populations that have a high risk of colon cancer. *Applied and Environmental Microbiology*.

[10] Gorbach SL, Tabaqchali S (1969). Bacteria, bile, and the small bowel. *Gut*.

[11] Simon GL, Gorbach SL (1986). The human intestinal microflora. *Digestive Diseases and Sciences*.

[12] Hill DA, Hoffmann C, Abt MC (2010). Metagenomic analyses reveal antibiotic-induced temporal and spatial changes in intestinal microbiota with associated alterations in immune cell homeostasis. *Mucosal Immunology*.

[13] Meyers JS, Ehrenpreis ED, Craig RM (2001). Small intestinal bacterial overgrowth syndrome. *Current Treatment Options in Gastroenterology*.

[14] Ivanov II, Atarashi K, Manel N (2009). Induction of intestinal Th17 cells by segmented filamentous bacteria. *Cell*.

[15] Wen L, Ley RE, Volchkov PYU (2008). Innate immunity and intestinal microbiota in the development of Type 1 diabetes. *Nature*.

[16] Eckburg PB, Bik EM, Bernstein CN (2005). Diversity of the human intestinal microbial flora. *Science*.

[17] Blaut M (2002). Relationship of prebiotics and food to intestinal microflora. *European Journal of Nutrition*.

[18] Itoh K, Lee WK, Kawamura H (1987). Intestinal bacteria antagonistic to Clostridium difficile in mice. *Laboratory Animals*.

[19] Rohde CL, Bartolini V, Jones N (2009). The use of probiotics in the prevention and treatment of antibiotic-associated diarrhea with special interest in *Clostridium difficile*—associated diarrhea. *Nutrition in Clinical Practice*.

[20] Taguchi H, Takahashi M, Yamaguchi H (2002). Experimental infection of germ-free mice with hyper-toxigenic enterohaemorrhagic *Escherichia coli* O157:H7, strain 6. *Journal of Medical Microbiology*.

[21] Gordon JI, Hooper LV, Shane McNevin M, Wong M, Bry L (1997). Epithelial cell growth and differentiation III. Promoting diversity in the intestine: conversations between the microflora, epithelium, and diffuse GALT. *American Journal of Physiology*.

[22] Umesaki Y, Okada Y, Matsumoto S, Imaoka A, Setoyama H (1995). Segmented filamentous bacteria are indigenous intestinal bacteria that activate intraepithelial lymphocytes and induce MHC class II molecules and fucosyl asialo GM1 glycolipids on the small intestinal epithelial cells in the ex-germ-free mouse. *Microbiology and Immunology*.

[23] Luciano L, Hass R, Busche R, Engelhardt WV, Reale E (1996). Withdrawal of butyrate from the colonic mucosa triggers ’mass apoptosis’ primarily in the G0/G1 phase of the cell cycle. *Cell and Tissue Research*.

[24] Ham M, Kaunitz JD (2007). Gastroduodenal defense. *Current Opinion in Gastroenterology*.

[25] Van der Sluis M, De Koning BAE, De Bruijn ACJM (2006). Muc2-deficient mice spontaneously develop colitis, indicating that MUC2 is critical for colonic protection. *Gastroenterology*.

[26] Kyo K, Muto T, Nagawa H, Lathrop GM, Nakamura Y (2001). Associations of distinct variants of the intestinal mucin gene MUC3A with ulcerative colitis and Crohn’s disease. *Journal of Human Genetics*.

[27] Al-Sadi R, Boivin M, Ma T (2009). Mechanism of cytokine modulation of epithelial tight junction barrier. *Frontiers in Bioscience*.

[28] Foti M, Ricciardi-Castagnoli P (2005). Antigen sampling by mucosal dendritic cells. *Trends in Molecular Medicine*.

[29] Telemo E, Korotkova M, Hanson LÅ (2003). Antigen presentation and processing in the intestinal mucosa and lymphocyte homing. *Annals of Allergy, Asthma and Immunology*.

[30] Himmel ME, Hardenberg G, Piccirillo CA, Steiner TS, Levings MK (2008). The role of T-regulatory cells and Toll-like receptors in the pathogenesis of human inflammatory bowel disease. *Immunology*.

[31] Hansen JJ, Holt L, Sartor RB (2009). Gene expression patterns in experimental colitis in IL-10-deficient mice. *Inflammatory Bowel Diseases*.

[32] Ostanin DV, Bao J, Koboziev I (2009). T cell transfer model of chronic colitis: concepts, considerations, and tricks of the trade. *American Journal of Physiology*.

[33] Yrios JW, Balish E (1986). Pathogenesis of Campylobacter spp. in athymic and euthymic germfree mice. *Infection and Immunity*.

[34] Martin HM, Campbell BJ, Hart CA (2004). Enhanced *Escherichia coli* adherence and invasion in Crohn’s disease and colon cancer. *Gastroenterology*.

[35] Baffy G (2009). Kupffer cells in non-alcoholic fatty liver disease: the emerging view. *Journal of Hepatology*.

[36] Van Egmond M, Van Garderen E, Van Spriel AB (2000). Fc*α*RI-positive liver Kupffer cells: reappraisal of the function of immunoglobulin A in immunity. *Nature Medicine*.

[37] Fox ES, Thomas P, Broitman SA (1989). Clearance of gut-derived endotoxins by the liver. Release and modification of 3H,14C-lipopolysaccharide by isolated rat Kupffer cells. *Gastroenterology*.

[38] Okumura Y, Ishibashi H, Shirahama M (1987). Kupffer cells modulate natural killer cell activity in vitro by producing prostaglandins. *Cellular Immunology*.

[39] Banchereau J, Briere F, Caux C (2000). Immunobiology of dendritic cells. *Annual Review of Immunology*.

[40] Kanto T, Hayashi N (2007). Innate immunity in hepatitis C virus infection: interplay among dendritic cells, natural killer cells and natural killer T cells. *Hepatology Research*.

[41] Gumperz JE (2004). CD1d-restricted "NKT" cells and myeloid IL-12 production: an immunological crossroads leading to promotion or suppression of effective anti-tumor immune responses?. *Journal of Leukocyte Biology*.

[42] Orange JS, Ballas ZK (2006). Natural killer cells in human health and disease. *Clinical Immunology*.

[43] Wisse E, Luo D, Vermijlen D, Kanellopoulou C, De Zanger R, Braet F (1997). On the function of pit cells, the liver-specific natural killer cells. *Seminars in Liver Disease*.

[44] Granucci F, Zanoni I, Pavelka N (2004). A contribution of mouse dendritic cell-derived IL-2 for NK cell activation. *Journal of Experimental Medicine*.

[45] Wiltrout RH, Herberman RB, Zhang SR (1985). Role of organ-associated NK cells in decreased formation of experimental metastases in lung and liver. *Journal of Immunology*.

[46] Radaeva S, Sun R, Jaruga B, Nguyen VT, Tian Z, Gao B (2006). Natural killer cells ameliorate liver fibrosis by killing activated stellate cells in NKG2D-dependent and tumor necrosis factor-related apoptosis-inducing ligand-dependent manners. *Gastroenterology*.

[47] Gao B, Radaeva S, Park O (2009). Liver natural killer and natural killer T cells: immunobiology and emerging roles in liver diseases. *Journal of Leukocyte Biology*.

[48] Li Z, Lin H, Yang S, Diehl AM (2002). Murine leptin deficiency alters Kupffer cell production of cytokines that regulate the innate immune system. *Gastroenterology*.

[49] Kremer M, Thomas E, Milton RJ (2010). Kupffer cell and interleukin-12-dependent loss of natural killer T cells in hepatosteatosis. *Hepatology*.

[50] Minagawa M, Deng Q, Liu Z-X, Tsukamoto H, Dennert G (2004). Activated natural killer T cells induce liver injury by Fas and tumor necrosis factor-*α* during alcohol consumption. *Gastroenterology*.

[51] Jiang W, Sun R, Zhou R, Wei H, Tian Z (2009). TLR-9 activation aggravates concanavalin A-induced hepatitis via promoting accumulation and activation of liver CD4^+^ NKT cells. *Journal of Immunology*.

[52] Crispe IN (2009). The liver as a lymphoid organ. *Annual Review of Immunology*.

[53] Roger P-M, Chaillou S, Breittmayer J-P (2005). Intrahepatic CD4^+^ T-cell apoptosis is related to METAVIR score in patients with chronic hepatitis C virus. *Scandinavian Journal of Immunology*.

[54] Zwacka RM, Zhang Y, Halldorson J, Schlossberg H, Dudus L, Engelhardt JF (1997). CD4^+^ T-lymphocytes mediate ischemia/reperfusion-induced inflammatory responses in mouse liver. *Journal of Clinical Investigation*.

[55] Safadi R, Ohta M, Alvarez CE (2004). Immune stimulation of hepatic fibrogenesis by CD8 cells and attenuation by transgenic interleukin-10 from hepatocytes. *Gastroenterology*.

[56] Takaku S, Nakagawa Y, Shimizu M (2003). Induction of hepatic injury by hepatitis C virus-specific CD8^+^ murine cytotoxic T lymphocytes in transgenic mice expressing the viral structural genes. *Biochemical and Biophysical Research Communications*.

[57] Lichtman SN, Keku J, Schwab JH, Sartor RB (1991). Hepatic injury associated with small bowel bacterial overgrowth in rats is prevented by metronidazole and tetracycline. *Gastroenterology*.

[58] Shomer NH, Fox JG, Juedes AE, Ruddle NH (2003). Helicobacter-induced chronic active lymphoid aggregates have characteristics of tertiary lymphoid tissue. *Infection and Immunity*.

[59] Moura SB, Mendes EN, Queiroz DMM (1999). Microbiological and histological study of the gastrointestinal tract of germ-free mice infected with Helicobacter trogontum. *Research in Microbiology*.

[60] Scott JR, Fox-Robichaud AE (2002). Hepatic leukocyte recruitment in a model of acute colitis. *American Journal of Physiology*.

[61] Navaneethan U, Shen B Hepatopancreatobiliary manifestations and complications associated with inflammatory bowel disease.

[62] John B, Crispe IN (2005). TLR-4 regulates CD8^+^ T cell trapping in the liver. *Journal of Immunology*.

[63] Numata Y, Tazuma S, Ueno Y, Nishioka T, Hyogo H, Chayama K (2005). Therapeutic effect of repeated natural killer T cell stimulation in mouse cholangitis complicated by colitis. *Digestive Diseases and Sciences*.

[64] Bambha K, Kim WR, Talwalkar J (2003). Incidence, clinical spectrum, and outcomes of primary sclerosing cholangitis in a united states community. *Gastroenterology*.

[65] Hines IN, Wheeler MD (2004). Recent advances in alcoholic liver disease III. Role of the innate immune response in alcoholic hepatitis. *American Journal of Physiology*.

[66] Bode C, Kugler V, Bode JC (1987). Endotoxemia in patients with alcoholic and non-alcoholic cirrhosis and in subjects with no evidence of chronic liver disease following acute alcohol excess. *Journal of Hepatology*.

[67] Uesugi T, Froh M, Arteel GE, Bradford BU, Thurman RG (2001). Toll-like receptor 4 is involved in the mechanism of early alcohol-induced liver injury in mice. *Hepatology*.

[68] Enomoto N, Ikejima K, Yamashina S (2001). Kupffer cell sensitization by alcohol involves increased permeability to gut-derived endotoxin. *Alcoholism: Clinical and Experimental Research*.

[69] Enomoto N, Ikejima K, Kitamura T (2000). Alcohol enhances lipopolysaccharide-induced increases in nitric oxide production by Kupffer cells via mechanisms dependent on endotoxin. *Alcoholism: Clinical and Experimental Research*.

[70] Wheeler MD, Kono H, Yin M (2001). The role of kupffer cell oxidant production in early ethanol-induced liver disease. *Free Radical Biology and Medicine*.

[71] Purohit V, Bode JC, Bode C (2008). Alcohol, intestinal bacterial growth, intestinal permeability to endotoxin, and medical consequences: summary of a symposium. *Alcohol*.

[72] Rao R (2009). Endotoxemia and gut barrier dysfunction in alcoholic liver disease. *Hepatology*.

[73] Fukui H, Kitano H, Okamoto Y (1995). Interaction of Kupffer cells to splenic macrophages and hepatocytes in endotoxin clearance: effect of alcohol. *Journal of Gastroenterology and Hepatology*.

[74] Ma TY, Nguyen D, Bui V, Nguyen H, Hoa N (1999). Ethanol modulation of intestinal epithelial tight junction barrier. *American Journal of Physiology*.

[75] Salaspuro M (1996). Bacteriocolonic pathway for ethanol oxidation: characteristics and implications. *Annals of Medicine*.

[76] Koppe SWP, Sahai A, Malladi P, Whitington PF, Green RM (2004). Pentoxifylline attenuates steatohepatitis induced by the methionine choline deficient diet. *Journal of Hepatology*.

[77] Satapathy SK, Sakhuja P, Malhotra V, Sharma BC, Sarin SK (2007). Beneficial effects of pentoxifylline on hepatic steatosis, fibrosis and necroinflammation in patients with non-alcoholic steatohepatitis. *Journal of Gastroenterology and Hepatology*.

[78] Diehl AM (2005). Lessons from animal models of NASH. *Hepatology Research*.

[79] Miele L, Valenza V, La Torre G (2009). Increased intestinal permeability and tight junction alterations in nonalcoholic fatty liver disease. *Hepatology*.

[80] Wu W-C, Zhao W, Li S (2008). Small intestinal bacteria overgrowth decreases small intestinal motility in the NASH rats. *World Journal of Gastroenterology*.

[81] Szabo G, Velayudham A, Romics L, Mandrekar P (2005). Modulation of non-alcoholic steatohepatitis by pattern recognition receptors in mice: the role of toll-like receptors 2 and 4. *Alcoholism: Clinical and Experimental Research*.

[82] Spruss A, Kanuri G, Wagnerberger S, Haub S, Bischoff SC, Bergheim I (2009). Toll-like receptor 4 is involved in the development of fructose-induced hepatic steatosis in mice. *Hepatology*.

[83] Miura K, Kodama Y, Inokuchi S, Schnabl B, Aoyama T, Ohnishi H Toll-like receptor 9 promotes steatohepatitis by induction of interleukin-1beta in mice.

[84] Wright RS, Anderson JW, Bridges SR (1990). Propionate inhibits hepatocyte lipid synthesis. *Proceedings of the Society for Experimental Biology and Medicine*.

[85] Cani PD, Bibiloni R, Knauf C (2008). Changes in gut microbiota control metabolic endotoxemia-induced inflammation in high-fat diet-induced obesity and diabetes in mice. *Diabetes*.

[86] Cani PD, Delzenne NM, Amar J, Burcelin R (2008). Role of gut microflora in the development of obesity and insulin resistance following high-fat diet feeding. *Pathologie Biologie*.

[87] Cani PD, Neyrinck AM, Fava F (2007). Selective increases of bifidobacteria in gut microflora improve high-fat-diet-induced diabetes in mice through a mechanism associated with endotoxaemia. *Diabetologia*.

[88] Hines IN, Hoffman JM, Scheerens H (2003). Regulation of postischemic liver injury following different durations of ischemia. *American Journal of Physiology*.

[89] Farhood A, McGuire GM, Manning AM, Miyasaka M, Smith CW, Jaeschke H (1995). Intercellular adhesion molecule 1 (ICAM-1) expression and its role in neutrophil-induced ischemia-reperfusion injury in rat liver. *Journal of Leukocyte Biology*.

[90] Shen X-D, Ke B, Zhai Y (2005). Toll-like receptor and heme oxygenase-1 signaling in hepatic ischemia/reperfusion injury. *American Journal of Transplantation*.

[91] Tsung A, Hoffman RA, Izuishi K (2005). Hepatic ischemia/reperfusion injury involves functional TLR4 signaling in nonparenchymal cells. *Journal of Immunology*.

[92] Devey L, Ferenbach D, Mohr E (2009). Tissue-resident macrophages protect the liver from ischemia reperfusion injury via a heme oxygenase-1-dependent mechanism. *Molecular Therapy*.

[93] Pillay SP, Wynter C, Lynch S, Wall D, Balderson G, Strong R (1997). Endotoxin levels in donors and recipients during orthotopic liver transplantation. *Australian and New Zealand Journal of Surgery*.

[94] Emre S, Sebastian A, Chodoff L (1999). Selective decontamination of the digestive tract helps prevent bacterial infections in the early postoperative period after liver transplant. *Mount Sinai Journal of Medicine*.

[95] Arai M, Mochida S, Ohno A, Arai S, Fujiwara K (1998). Selective bowel decontamination of recipients for prevention against liver injury following orthotopic liver transplantation: evaluation with rat models. *Hepatology*.

[96] Lee J-S, Thorgeirsson SS (2004). Genome-scale profiling of gene expression in hepatocellular carcinoma: classification, survival prediction, and identification of therapeutic targets. *Gastroenterology*.

[97] Groopman JD, Kensler TW, Wild CP (2008). Protective interventions to prevent aflatoxin-induced carcinogenesis in developing countries. *Annual Review of Public Health*.

[98] Romeo R, Colombo M (2002). The natural history of hepatocellular carcinoma. *Toxicology*.

[99] Lopez JB (2005). Recent developments in the first detection of hepatocellular carcinoma. *The Clinical Biochemist. Reviews*.

[100] Fox JG, Feng Y, Theve EJ (2010). Gut microbes define liver cancer risk in mice exposed to chemical and viral transgenic hepatocarcinogens. *Gut*.

[101] Abu Al-Soud W, Stenram U, Ljungh A, Tranberg K-G, Nilsson H-O, Wadström T (2008). DNA of *Helicobacter* spp. and common gut bacteria in primary liver carcinoma. *Digestive and Liver Disease*.

[102] Xuan S-Y, Li N, Qiang X, Zhou R-R, Shi Y-X, Jiang W-J (2006). Helicobacter infection in hepatocellular carcinoma tissue. *World Journal of Gastroenterology*.

[103] Bataller R, Brenner DA (2005). Liver fibrosis. *Journal of Clinical Investigation*.

[104] Seki E, De Minicis S, Österreicher CH (2007). TLR4 enhances TGF-*β* signaling and hepatic fibrosis. *Nature Medicine*.

[105] Shi Z, Wakil AE, Rockey DC (1997). Strain-specific differences in mouse hepatic wound healing are mediated by divergent T helper cytokine responses. *Proceedings of the National Academy of Sciences of the United States of America*.

[106] Isayama F, Hines IN, Kremer M (2006). LPS signaling enhances hepatic fibrogenesis caused by experimental cholestasis in mice. *American Journal of Physiology*.

[107] Paik Y-H, Lee KS, Lee HJ (2006). Hepatic stellate cells primed with cytokines upregulate inflammation in response to peptidoglycan or lipoteichoic acid. *Laboratory Investigation*.

[108] Paik Y-H, Schwabe RF, Bataller R, Russo MP, Jobin C, Brenner DA (2003). Toll-like receptor 4 mediates inflammatory signaling by bacterial lipopolysaccharide in human hepatic stellate cells. *Hepatology*.

[109] Bauer TM, Steinbrückner B, Brinkmann FE (2001). Small intestinal bacterial overgrowth in patients with cirrhosis: prevalence and relation with spontaneous bacterial peritonitis. *American Journal of Gastroenterology*.

[110] Gunnarsdottir SA, Sadik R, Shev S (2003). Small intestinal motility disturbances and bacterial overgrowth in patients with liver cirrhosis and portal hypertension. *American Journal of Gastroenterology*.

[111] Miettinen TA (1972). Lipid absorption, bile acids, and cholesterol metabolism in patients with chronic liver disease. *Gut*.

[112] Sung JY, Shaffer EA, Costerton JW (1993). Antibacterial activity of bile salts against common biliary pathogens. Effects of hydrophobicity of the molecule and in the presence of phospholipids. *Digestive Diseases and Sciences*.

[113] Inagaki T, Moschetta A, Lee Y-K (2006). Regulation of antibacterial defense in the small intestine by the nuclear bile acid receptor. *Proceedings of the National Academy of Sciences of the United States of America*.

[114] Castilla-Cortázar I, Pascual M, Urdaneta E (2004). Jejunal microvilli atrophy and reduced nutrient transport in rats with advanced liver cirrhosis: improvement by insulin-like growth factor I. *BMC Gastroenterology*.

[115] Washington MK (2007). Autoimmune liver disease: overlap and outliers. *Modern Pathology*.

[116] Kremer M, Perry AW, Milton RJ, Rippe RA, Wheeler MD, Hines IN (2008). Pivotal role of Smad3 in a mouse model of T cell-mediated hepatitis. *Hepatology*.

[117] Jaruga B, Hong F, Sun R, Radaeva S, Gao B (2003). Crucial role of IL-4/STAT6 in T cell-mediated hepatitis: up-regulating eotaxins and IL-5 and recruiting leukocytes. *Journal of Immunology*.

[118] Kondo T, Suda T, Fukuyama H, Adachi M, Nagata S (1997). Essential roles in the Fas ligand in the development of hepatitis. *Nature Medicine*.

[119] Seino K-I, Kayagaki N, Takeda K, Fukao K, Okumura K, Yagita H (1997). Contribution of Fas ligand to T cell-mediated hepatic injury in mice. *Gastroenterology*.

[120] Takeda K, Hayakawa Y, Van Kaer L, Matsuda H, Yagita H, Okumura K (2000). Critical contribution of liver natural killer T cells to a murine model of hepatitis. *Proceedings of the National Academy of Sciences of the United States of America*.

[121] Mizuhara H, Uno M, Seki N (1996). Critical involvement of interferon gamma in the pathogenesis of T-cell activation-associated hepatitis and regulatory mechanisms of interleukin-6 for the manifestations of hepatitis. *Hepatology*.

[122] Hopf U, Stemerowicz R, Rodloff A (1989). Relation between *Escherichia coli* R(rough)-forms in gut, lipid A in liver, and primary biliary cirrhosis. *Lancet*.

[123] Silveira MG, Lindor KD (2008). Primary sclerosing cholangitis. *Canadian Journal of Gastroenterology*.

[124] Wojczys R (1997). Liver involvement and its course in experimental colitis in rats. *Hepato-Gastroenterology*.

[125] Fung J, Lai C-L, Yuen M-F (2009). Hepatitis B and C virus-related carcinogenesis. *Clinical Microbiology and Infection*.

[126] Perrault M, Pécheur E-I (2009). The hepatitis C virus and its hepatic environment: a toxic but finely tuned partnership. *Biochemical Journal*.

[127] Machida K, Tsukamoto H, Mkrtchyan H (2009). Toll-like receptor 4 mediates synergism between alcohol and HCV in hepatic oncogenesis involving stem cell marker Nanog. *Proceedings of the National Academy of Sciences of the United States of America*.

